# Relatives’ Perspectives on What Works to Reduce Problematic Alcohol Use in Older Adults: A Realist Evaluation

**DOI:** 10.1007/s11469-025-01511-4

**Published:** 2025-07-14

**Authors:** Fieke A. E. van den Bulck, Andrea D. Rozema, Rob H. L. M. Bovens, Rikste Knijff, Dike van de Mheen, Rik Crutzen

**Affiliations:** 1 Tranzo Scientific Center for Care and Wellbeing, School of Social and Behavioral Sciences, Tilburg University, Tilburg, The Netherlands; 2 Positive Lifestyle Foundation, Nijmegen, The Netherlands; 3 Department of Health Promotion, Care and Public Health Research Institute (CAPHRI), Maastricht University, Maastricht, The Netherlands

**Keywords:** Realist evaluation, Relatives, Older adults, Intervention, Alcohol

## Abstract

The rising prevalence of problematic alcohol use among older adults poses challenges to their health and also impacts their relatives. Given the important role relatives play, the objective of this study was to explore their perspectives on working elements (E) and mechanisms (M) within different contexts (C), both within and outside interventions, contributing to the outcome (O) of reducing (problematic) alcohol use among older adults. Guided by a realist evaluation approach, an existing initial program theory (IPT) on working elements in alcohol interventions was tested by conducting interviews with older adults’ partners and family members (*N* = 14). Data was coded and links between contexts, elements, mechanisms, and outcomes (CEMOs) were sought for to confirm, refute, or refine the IPT. In different contexts, several working elements were identified: (1) paying attention to the causes of use, underlying issues and established patterns, (2) receiving a personalized approach, (3) receiving feedback, (4) having a clear structure in program and meetings, (5) having frequent contact with practitioner and/or peers, (6) hearing the impact of alcohol use on relatives, and (7) relatives’ involvement in interventions and abstinence. Two important mechanisms were having a measure of control and having a deterrent to drink. Both as an addition to the IPT and from the viewpoint of relatives, there is a need to focus on confrontation, involvement of relatives in abstinence and interventions, having structure and contact, and having a measure of control and a deterrent to drink, to reduce problematic alcohol use in older adults.

**Supplementary Information:**

The online version contains supplementary material available at 10.1007/s11469-025-01511-4.

Problematic alcohol consumption among older adults is growing and is associated with negative consequences for these people (Breslow et al., 2017; Kerr et al., 2024; Wolf et al., 2017). These consequences include increased risk of falling and prolonged and potentially worsened health effects due to slower alcohol metabolism in older adults (Kinirons & O’Mahony, 2004; Meier & Seitz, 2008; Rubenstein, 2006). There are also difficulties experienced by relatives in the presence of older adults’ problematic alcohol use. For example, older adults’ alcohol use can negatively affect their children’s alcohol use and wellbeing. Additionally, spouses of older adults with alcohol-related problems often experience more health issues, emotional problems and depressive symptoms and they tend to be less involved in social activities (Johannessen et al., 2022; Moos et al., 2010b; Sacco et al., 2009).

Relatives play an important role in abstinence among older adults. On the one hand, relatives can influence older adults’ abstinence and treatment negatively, for example when they cause stress, or strongly approve and encourage older adults’ drinking (Holland et al., 2016; Moos et al., 2010a, b). On the other hand, relatives can be a source of support for older adults. For example, concerns expressed by older adults’ family members and friends about their drinking behavior can influence the likelihood of seeking treatment (Room et al., 2004). Furthermore, a family-oriented approach to interventions appears to be helpful for older adults with problematic alcohol use (Stelle, 2007).

Given the importance of the role the relatives play in older adults’ alcohol use, it is warranted to take their perspective to gain a holistic understanding of what works to reduce problematic alcohol use in and around interventions. Such a holistic understanding is beneficial because it can unravel working elements and mechanisms that may be overlooked by clients and professionals. Therefore, it is important to explore what is already known about what works for older adults and then examine the perspectives of relatives as well.

A previous systematic review focused on understanding how (working elements (E)), in which context (C), and why (mechanisms (M)) interventions are successful in reducing (problematic) alcohol use among adult people in general, and older adults specifically (outcomes (O)) (Boumans et al., 2022). Working elements in six contexts were identified. In various contexts, the review identified three general working elements: (1) providing information about the consequences of alcohol consumption; (2) personalized feedback about drinking behavior; and (3) being in contact with others and communicating with them about (alcohol) problems. Of the 61 studies, only 3 evaluated interventions for older adults, and only the first and second general working elements were found in these studies.

Two studies expanded upon this review and explored the perspectives of professionals providing interventions and older adults participating in interventions regarding working elements and mechanisms in interventions (Van den Bulck et al., 2024, 2025). Remarkably, both professionals and older adults identified social contact and support from relatives as general working elements, while these were not identified in the three studies focusing on older adults within the previous mentioned review (Boumans et al., 2022).

The objective of this study is to explore the working elements (E) and mechanisms (M) in interventions within different contexts (C), both within and outside interventions, contributing to the outcome (O) of reducing (problematic) alcohol use among older adults, from the perspective of relatives of older adults participating in interventions.

This study builds on the previously mentioned systematic review (Boumans et al., 2022), using a realist evaluation (RE) approach. RE examines how interventions and their elements (E) work differently across contexts (C), because the mechanisms (M) needed for successful interventions outcomes (O) are activated to a different extent. RE unpacks the relationships between these constructs, which is called the context-element-mechanism-outcome (CEMO) configuration (Pawson et al., 1997). RE starts with formulating an initial program theory (IPT) based on CEMOs found in literature. The findings in six different interventions contexts of the previously mentioned review’s findings (Boumans et al., 2022) form our IPT and will be tested in this study among relatives to confirm, refute, or refine the CEMOs of the IPT.

## Method

### Study Design

Semi-structured interviews with a RE approach were conducted with relatives, i.e., partners or family members, of older adults who participated in interventions to reduce their (problematic) alcohol use. This study is part of a larger project, also investigating the perspectives of professionals and older adults (Van den Bulck et al., 2024, 2025). This study received approval by the Ethics Review Board (RP508) of Tilburg University. The COREQ guidelines were followed in reporting (Tong et al., 2007), (see Supplementary Material 1). All included participants read an informed consent form and gave oral informed consent prior to the interviews.

### Sampling and Recruitment

We recruited relatives of older adults who had participated in interventions for their alcohol use, which is why interventions serve as the link for participant recruitment. We included interventions if they (1) focused on preventing or reducing (problematic) alcohol use; (2) were provided in an individual and/or group setting with or without relative involvement; (3) were provided face to face, online, and/or via telephone; (4) were provided in one country, because the functioning of interventions could be influenced by national healthcare policies, or cultural and social factors; and (5) were provided within the last two years. Interventions were excluded if they (1) were not primarily aimed at lifestyle change; (2) were provided in clinical admission; (3) were group-specific interventions (e.g., for veterans); (4) focused on general health and lifestyle improvements without explicit mention of alcohol use; (5) focused solely on recovery; or (6) focused on someone other than the client with problematic alcohol use (e.g., only on relatives).

To find relevant interventions, we searched in the Dutch National Database Centre for Healthy Living, consulted national working groups on alcohol and older adults, and reached out to professionals via our network, LinkedIn, and online support groups.

To recruit relatives, we approached 20 older adults who participated in the larger research project that investigated the perspectives of professionals and older adults (Van den Bulck et al., 2025), 39 professionals from organizations working with older adults and relatives, and members of online support groups. We, or the professionals who assisted with recruitment, sent the older adults an information letter with an invitation for their relatives to participate, or, if possible, sent it directly to the relatives. In total, 14 relatives participated. Participant demographic characteristics are shown in Table 1.

**Table 1:** Participants’ demographic characteristics

Baseline characteristic	Participants
	n = 14*
Age (years)	
30–34	2
55–64	4
65–74	6
≥ 75	1
Gender	
Male	9
Female	5
Relationship to person received intervention	
Partner	10
Child	3
Sibling	1
Education level**	
Lower education	1
Intermediate education	7
Higher education	5
Intervention***	
AA	3
Fresh Onward	1
Moti-55	1
NoThanks	3
Online support group	1
Other: brief interventions, and treatment in mental health or addiction organization	3
Schema therapy	1
SoberCare	3

*One participant’s demographics (age and educational level) were missing

**Lower education = Primary education; the first three years of senior general secondary education (HAVO) and pre-university secondary education (VWO); prevocational secondary education (VMBO); lower secondary vocational training and assistant’s training (MBO-1), Intermediate education = Upper secondary education (HAVO/VWO), basic vocational training (MBO-2), vocational training (MBO-3), and middle management and specialist education (MBO-4), Higher education = Higher vocational education (HBO), university education (WO)

***Some older adult clients participated in more than one intervention

### Data Collection

Based on the participants’ preferences, interviews were conducted face to face (*n* = 1), online (*n* = 1) or by telephone (*n* = 12). Interviews were performed between June 2022 and March 2024, each lasting an average of 55.2 (range: 32.6**–**70.1) minutes. We used an interview guide (see Supplementary Material 2) that measured, first inductively, the perceived outcomes of the interventions (O), and what working elements (E) and mechanisms (M) contributed to these outcomes, and the influence of the contexts (C) in which the interventions were provided. Since we noticed that participants sometimes had limited insight into the content of the intervention or its working elements, we decided to also ask about working elements and mechanisms in the context beyond participation in the intervention, which could contribute to the outcome of abstinence. An example of a context beyond participation is the home setting, where the working element interacts with family members. At the end of the interview guide, we presented the previously found CEMO configurations of the IPT of the review of Boumans et al. (2022); we invited participants to confirm, refute, or refine the CEMOs.

### Data Analysis

Professional transcriptionists transcribed the interviews verbatim. FAEvdB drafted a code tree based on the IPT that encompassed the CEMO configurations. The IPT consisted of six combinations of three intervention contexts: (1) whether the client was in contact with a practitioner; (2) whether the intervention was provided in-person; and (3) regarding individual treatment, group treatment, or treatment with relatives’ involvement. However, in the current study, two intervention contexts were added in the program theory since we collected data in two contexts beyond the IPT. Elements that were not linked to a specific intervention context were categorized as “general,” while those mentioned as supplementary or outside the intervention were placed in the context “not in intervention.”

Independently and in parallel, FAEvdB and RK coded two transcripts, focusing on identifying links between contexts, elements, mechanisms, and outcomes (Pawson et al., 1997). After coding the first transcript, FAEvdB cross-checked RK's configurations and coding. FAEvdB and RK discussed any discrepancies in configurations and made refinements where needed. This process was repeated for the two interviews until coding alignment was reached. Thereafter, FAEvdB independently coded the remaining interviews. When uncertainty arose regarding coding for specific data sections, FAEvdB consulted with RK. To further ensure the dependability of the coding, RC and RK reviewed the codes and CEMOs iteratively and provided feedback. The data were analyzed using Atlas.TI.

## Results

Results could be divided into eight themes of working elements based on the current findings: (1) paying attention to drinking behavior, (2) paying attention to lifestyle and activities, (3) the relationship between the client and practitioner, (4) the relationship between the client and peers, (5) the relationship between the client and relatives, (6) communication and approach, (7) setting, and (8) individual elements. Table 2 provides the program theory with an overview of the working elements (E), mechanisms (M), outcomes (O), and contexts (C).

**Table 2:** The program theory

Working element (E)	Mechanism (M)		Outcome (O)	Context (C)						IPT ***
				In intervention **					Not in intervention	
				General	Individual		In group			
					No practitioner	Practitioner	No practitioner	Practitioner		
1. Paying attention to drinking behavior										
Paying attention to drinking behavior and abstinence	Thinking about alcohol use		Less or no alcohol use			X	X†			✓
	Realization of doing wrong									
Conversation with practitioner about alcohol use and solutions	Having a wake up call for change			X				X		+
	Gaining new insights to approach problematic use									
Paying attention to cause, underlying problems and traumas			Less or no alcohol use	X		X		X		+
Tracking alcohol use and reflecting on use and progress abstinence	Awareness alcohol use			X		X				+
Daily or regular breath testing and explain yourself about it	Exerting control and pressure		No alcohol use			X†				+
Participating in an abstinence challenge	Creating awareness				X†					+
Experiencing abstinence and fitness			Less alcohol use	X					X	+
2. Paying attention to lifestyle and activities										
Motivating to change lifestyle (in a work setting)										~
Paying attention to lifestyle	Avoiding to introspect, that could lead to depression		Less or no alcohol use					X		+
Doing volunteer work or engage in other daytime activities	Having a deterrent to drink the day before		Less or no alcohol use						X	+
	Because otherwise you'll start to introspect, which can lead to depression									
Engaging in substitute or distracting activities to avoid drinking alcohol	Having less temptation from alcohol at home		Less or no alcohol use		X†				X	+
	Gaining motivation									
3. The relationship between client and practitioner										
Practitioner has personal life experience and experiential knowledge about problematic alcohol use	Practitioner has better understanding of client’s life stage issues	Becoming more open				X		X		+
	Practitioner has better understanding of the client									
	Being open to seeking help for problems									
Practitioner is a good dialogue leader								X		+
Practitioner provides advice and practical tools			No alcohol use			X, X†		X		+
Practitioner asks probing questions				X						+
Practitioner is empathetic	Collaborating in the identification of help and needs, and improving the relationship between the client and practitioner			X		X				✓
The client feels seen and heard in interactions with practitioner	Feeling less loneliness		Less or no alcohol use	X		X				+
	Feeling free to share a story that relatives have sometimes heard 20 times already									
Practitioner confronts, speaks firmly, and holds up a mirror	Being confronted	Realizing that change is needed	Less or no alcohol use	X		X, X†				
4. The relationship between client and peers										
Contact with and presence of peers with same level of problematic alcohol use	Creating a sense of togetherness		Less or no alcohol use	X			X†	X		+
	Feeling understood									
	Recognizing that you are not alone									
Client feels seen and heard by peers			No alcohol use	X				X		+
Sharing experiences and tips with peers				X			X, X†	X, X†		+
Making agreements together with peers							X		X	+
Trust, respect, and confidentiality in the group				X						+
5. The relationship between client and relatives										
Contact with and presence of relatives	Having a deterrent to drink			X					X	+
Openness in the environment about alcohol use and participation in an intervention	Being able to share your story with environment								X	+
	Feeling understood, accepted, and better understood									
	People can address client									
Relatives participate in abstinence, and abstinent environment									X	+
Involving relatives in the intervention	Relatives can provide support to client			X						+
Discussing the reasons and causes of problematic use with relatives and gaining insight into established patterns	Gaining insights into each other and feeling heard	Having a deterrent to drink	No alcohol use	X		X‡	X†			+
	Realizing that there is benefit to be gained by doing things differently									
Collaboration with relatives to develop a plan of action			Less or no alcohol use	X						+
Relatives are informed about the intervention				X						+
Relatives learn about the client and problematic alcohol use	Relatives recognize the problems and better understand the client			X						+
Relatives learn how to deal with alcohol use of client	Relatives better understand and support the client		Less or no alcohol use			X‡				✓
	Relatives have more insight and empathy towards the client, and the relative can provide better support									
	Being more open to criticism from relatives									
Relatives monitor/control the client				X		X†, X‡				+
Relatives encourage and put pressure	Because family is important, the client will listen to them and be motivated to seek help		Less alcohol use						X	+
Client has to account to relatives about alcohol and abstinence	Having a controlling function around		No alcohol use			X†				+
Client hears what alcohol use does to relatives	Recognition of the problem and	Being open to seeking help for it				X, X†		X†		+
	More understanding for relatives’ issues	Making it easier for the client to decide to stop drinking								
Relatives address and confront the client, and learn how to do this						X‡			X	+
Relative shares their experience with the client’s use	Gaining insight into the impact of use	Having a deterrant to not relapse	Less or no alcohol use			X‡				+
Joint and supportive approach from relatives	Supporting each other					X†			X	+
	Having a greater chance of overcoming problematic use									
	Having a social safety net									
6. Content and approach										
App	Gaining insight into alcohol use		Less or no alcohol use		X†					+
Personal contact and feedback										~
Online communication and feedback										~
Personal approach	Awareness		Less or no alcohol use			X, X†		X		+
Receiving feedback	Gaining motivation			X		X†		X		+
Homework exercises	Having more engagement and being provided with tools					X		X		+
	Awareness									
7. Setting										
Web-based and telephone-based interventions										~
Online participation	Allowing to participate from home in a safe environment			X	X†					+
	Easy and low-threshold participation									
	Not having to make yourself vulnerable to others									
Anonymous participation	Easy and low-threshold participation	Motivation to participate			X†	X†				+
	Low-threshold participation									
Frequent contact	Having commitment and accountability		Awareness and regulating alcohol use			X, X†	X†	X		+
			No alcohol use							
	Having regularity									
	Having a deterrant not to relapse									
	Exerting control									
Intensive and active participation	Providing regularity		No alcohol use		X†			X		+
Program and meetings have a clear structure	Having a clear understanding of what is expected		No alcohol use	X		X†	X†			+
8. Individual elements										
Motivation									X	+
Willpower									X	+
Confrontation				X						+

*Elements of Initial program theory that were refined, or were not confirmed

** † = not face to face, ‡ with relative involvement

***IPT ✓ = Initial program theory confirmed, ~ = Initial program theory refined, + = Program theory added − = Initial program theory not confirmed

## Paying Attention to Drinking Behavior

Different forms of attention related to alcohol use were mentioned as important, namely *paying attention to alcohol use and abstinence* (E), as well as for the *cause, underlying problems, and traumas* (E). Actively engaging with alcohol use was also believed to help reducing alcohol use, e.g., by *tracking alcohol use* (E), *experiencing abstinence* (E), *participating in an abstinence challenge* (E), and doing *daily or regular breath testing* (E).

## Paying Attention to Lifestyle and Activities

Participants said that *paying attention to lifestyle* (E) in interventions (C) could help clients to reduce their alcohol use or stop drinking. Outside of interventions (C), *volunteering or engaging in other daytime activities* (E), or keeping oneself occupied with *substitute or distracting activities* (E), helps because doing such activities would be motivating (M).

Hobbies and such, so that you’re not sitting at home every day with nothing to do. You do need something to fill your day, so you can enjoy yourself. And that’s not easy, because it is so easy to reach for a bottle and then you feel good. But then you just feel awful because you haven’t done anything. So that lethargy, it’s terrible for older people. [R3]

## The Relationship Between the Client and Practitioner

Participants mentioned that the practitioner plays an important role in interventions. First, expertise and experience were crucial, i.e., *personal life experience and experiential knowledge about problematic alcohol use* (E), because this fostered greater understanding (M) for the client, and the *ability to provide good advice and guidance* (E). The practitioner also needed to have certain communication skills, including the ability to *ask probing questions* (E), and to *confront when necessary* (E). Social skills were also mentioned, particularly an *empathic approach* (E), and ensuring *the client feels seen and heard in interactions* (E).

If you have experienced something yourself that your client has also gone through, then you can empathize better. [...] That he (the practitioner) is an expert by experience with alcohol [...] Interviewer: What does that do for the participant? Participant: Recognition and maybe also a sense of: you’re not the only one... a bit of support. [R5]

## The Relationship Between Client and Peers

In group interventions (C), *the contact with and presence of peers with a similar level of problematic alcohol use* (E) was important because it fostered a sense of belonging (M), recognition that one is not alone (M), and understanding, as peers often go through similar experiences (M).

When you’re around people who don’t have that problematic alcohol use... they think: “I understand it”. But they don’t really understand, because they don’t have that problem. So I think it’s important that you see people who have the same problem. Then you can talk about it and you understand each other. And other people who don’t have that problem, they just really don’t understand. They might understand a little, but it’s still different. [R10]

More specifically, it was helpful in their interactions to *share experiences and tips* (E) and to *make agreements together* (E). In their relationships, it was important for the client to *feel seen and heard* (E), and for there to be *trust, respect, and confidentiality* (E).

You’re sharing something, and the reason why you share things can be different, but there’s always psychological suffering or a psychological problem underlying it, and that is shared and recognized. I think that creates a bond between the participants, as well as empathy and care for each other […] so you develop a sense of solidarity from it. I also believe it supports the collective principle because you’re less likely to relapse. [R11]

## The Relationship Between Client and Relatives

According to the participants themselves, they played an important role in reducing or stopping the client’s drinking. Both during and outside interventions, their involvement was important, including the *contact with relatives and their presence* (E), *relatives’ participation in abstinence efforts* (E), *involvement and collaboration in interventions* (E), such as developing a plan of action for the client, and *relatives being informed about the intervention’s content* (E). Also, communication and openness with relatives were important, i.e., *being open about alcohol use and participation in an intervention* (E), and *discussing the reason and causes of problematic use with relatives* (E). Education for relatives was also helpful to reduce drinking, such as *teaching the relative about the client and problematic alcohol use* (E) and *teaching to manage client’s alcohol use* (E), as these elements contributed to greater understanding (M) and better support (M) for the client. Relatives also mentioned that that *controlling* (E), *encouraging* (E), and *applying pressure on the client* (E) could help reduce drinking and that *the client must be held accountable to their relatives for their alcohol use* (E).

She retired early, and that was a reason for me to say: “If you don’t have that under control before you retire, then I don’t see a future for us anymore, because I’m not going to go out with a drunk woman every day.” And I think that pressure from me—I don’t know if I handled that well […] —I think that pressure contributed to her trying to stay abstinent. [R7]

They also noted that *confrontation by relatives* (E) and *hearing how alcohol use affects relatives* (E) are effective for clients, as they provide insight into the impact of alcohol use (M) and help the client recognize its problematic nature (M).

It certainly served as a deterrent, when I looked my father straight in the eyes and told him: “It really affected me and shaped me into who I am now”. And then he naturally became emotional and he really realized: “Wow, I didn’t bully my children, but I did hurt them in that regard, and that’s truly, truly the realization that I’m never going to do that again. I never want to relapse like that again.” [R6]

## Content and Approach

Participants mentioned that *feedback* (E) can help reduce alcohol use because it can foster motivation (M). Moreover, they indicated that in interventions involving a practitioner (C), it helps to have a *personalized approach* (E) and have *homework exercises* (E), as both could raise awareness (M). Lastly, a *telephone app* (E) could also contribute to reducing alcohol use, as it could provide insight into alcohol use (M).

## Setting

Both *online* and *anonymous participation* (E) were mentioned as working elements in individual interventions (C). Additionally, in the presence of others, i.e., contact with a practitioner and/or group setting (C), *frequent contact* (E) was highlighted as effective because it fosters commitment (M), accountability (M), control (M), and serves as a deterrent to start drinking (M). Lastly, *intensive and active participation* (E) was important, along with a *clear structure for the program and meetings* (E).

## Individual Elements

In addition to participating in an intervention (C), participants considered *motivation* (E) and *willpower* (E) to be important for clients. *Confrontation* (E) was also mentioned as a key trait.

## Discussion

### Key Findings

Overall, seven elements that were mentioned in minimally three contexts by more than three participants were identified as general working elements: (1) *paying attention to the causes of use, underlying issues, and established patterns*, (2) *receiving a personalized approach, *(3) *receiving feedback,* (4) *having a clear structure in program and meetings*, (5) *having frequent contact with practitioner and/or peers*, (6) *hearing the impact of alcohol use on relatives*, and (7) *relatives’ involvement in interventions and abstinence*. We also found working elements mentioned by more than three participants in specific contexts, namely (a) the practitioner: *has life experience and experiential knowledge about problematic alcohol use*, *provides advice and tools*, and *confronts, speaks sternly, and holds up a mirror*; (b) the group setting includes *contact with and presence of peers*, and *sharing experiences and tips with peers*; and (c) *anonymous participation* in an individual setting. Moreover, two mechanisms noted by more than three participants and prevalent in multiple contexts are having a measure of *control* and a *deterrent.*

### Interpretation of Findings

The first addition to the IPT was the importance of confronting one’s own alcohol use and its consequences, which could be done by both a practitioner and a relative. Earlier findings on the effectiveness of confrontation are mixed, with some suggesting it may be counterproductive, while others indicate potential benefits. Practitioner confrontation does not always seem to contribute to positive intervention outcomes (Karno, 2007; Polcin et al., 2006), and older adults themselves appear to prefer a non-confrontational approach by therapists (Holland et al., 2016). Confrontation by relatives can lead to resistance if it is carried out in a hostile or overly aggressive manner. Confrontations lacking trust, caring, and respect in the relationship can also have a negative impact, often resulting in the individual feeling the urge to rebel against the advice being offered (Polcin et al., 2012). Nonetheless, confrontation from family and friends can also be perceived as supportive and helpful, especially among individuals with higher levels of substance use and those who believed it would be difficult to remain sober (Polcin, 2009). The way in which confrontation is handled may determine whether it has positive effects. Open communication can help families in confronting concerns and convey feelings, thereby reducing miscommunication and the potential for conflict (Rangarajan & Kelly, 2006). Additionally, confrontations are more effective when they include offers of practical help, encouragement, and a sense of hope, rather than being delivered in an aggressive manner (Polcin et al., 2012).

The second addition to the IPT was the importance of relatives’ involvement in the intervention and abstinence. Involvement in interventions include physical presence, informing them about the program’s content, and involving them in a plan of action for the client. The importance of involvement aligns with other research (Tyo & McCurry, 2023), with one study also highlighting that family members experienced frustration with a mental health/addictions system that excluded them from treatment decisions and with the general lack of information and support for family members. Help for the client could be improved if family members’ insights were taken into consideration in treatment planning (Haskell et al., 2016). Additionally, involving family members in treatment has been found to be effective: behavioral family counseling for substance abuse, where family members participate in the treatment, leads to better retention of individuals in treatment, improved drug and alcohol abstinence, and enhanced relationship adjustment (O’Farrell et al., 2010).

The relatives’ involvement in abstinence related to the role they played in the older adult’s abstinence, i.e., by being abstinent themselves, encouraging the client, and addressing them when necessary. Prior research already demonstrated that the social environment plays an important role in alcohol use among older adults. For instance, drinking behaviors of older married couples are influenced by their spouses (Polenick et al., 2018; Reczek et al., 2016), and family and friends can influence both the frequency and quantity of older adults, either by promoting or regulating it (Vogelsang & Lariscy, 2020). Moreover, marital quality may also play an important role, as alcohol use may increase in positive relationships (Bulanda et al., 2023). Given the important role relatives play, it is crucial to effectively leverage their involvement. For example, understanding the dynamics within relationships can help address potential conflicts or barriers to abstinence.

The third addition to the IPT was having structure and contact. This relates both to the intervention itself, i.e., having a clear structure in the intervention and frequent contact with a practitioner and/or peers, as well as outside of the intervention, i.e., engaging in daytime activities. Having structure in everyday life has been associated with less alcohol use (Crutzen & Knibbe, 2012). This might be particularly important for older adults after retirement, as they can lose social roles that provide fulfillment. Retirement can also lead to increased alcohol use due to more leisure time and opportunities for socialization (Bareham et al., 2019; Nicholson et al., 2017), although reduced occasions for socialization may, on the other hand, lead to a decrease in alcohol consumption (Dare et al., 2014; Haarni & Hautamäki, 2010).

Moreover, two mechanisms that were mentioned by more than three participants were found in multiple contexts: having a measure of control and having a deterrent to drink. Both focus on constraints, although control involves more active oversight or regulation by others, while deterrent to drink is more about discouraging behavior through indirect influences.

Having a measure of control arose in interactions between the client and relatives or those present in an intervention, where the client had to justify or account for their alcohol use to others, or relatives who monitored the client. Family indeed seems to play an important role in treatment, including the pressure they can exert on older adults to enter treatment (Emiliussen et al., 2017). Once in treatment, the roles of control and pressure appear to have a dual nature. Relatives may express a desire for more control and support for the client, yet, recovery-oriented approached emphasize the critical role of agency and self-determination on the part of the client (Onken et al., 2007). Additionally, clients may sometimes prefer a style that allows them to maintain their independence rather than having intensive support and control (Noble & Douglas, 2004).

Having a deterrent to drinking also arose in interactions with relatives. The mere presence of relatives, rather than the content of the interactions, played a significant role. Alongside their presence, participation in physical daytime activities and volunteer work served as a deterrent to drink.

### Limitations

Many clients were not open to involving their relatives in the study, which may have led to a limited representation of relatives of clients who keep their problematic use secret from their relatives, since the recruitment of relatives mainly occurred through older adult clients who participated in interventions. Recruitment through other platforms, such as family support organizations, contributed to a broader representation of relatives. Furthermore, relatives were not always aware of the exact content of the interventions. Although they were specifically asked about what they thought were working elements and mechanisms, their response was sometimes hypothetical in nature or mostly based on assumptions. This speculative interpretation may have resulted in less concrete insights into the CEMOs. The results should be interpreted considering that only a limited number of relatives were interviewed, which may not be representative of the broader population of relatives.

### Implication

An important implication of this study is that relatives’ perspectives should be considered alongside those of professionals and clients. While relatives offer valuable insights from the home environment, these must be balanced with the expertise of professionals and clients’ experiences for a complete view of working elements and mechanisms in interventions. We also recommend further research into the needs of relatives. Stigma and self-blame are common among relatives of older adults with problematic substance use, and some families cope with stigma by remaining silent about the substance use problem (O’Shay-Wallace, 2020; Sarno and et al., 2021). Understanding these dynamics is important for interventions to effectively support both older adults and their relatives’ needs.

## Conclusion

In addition to the IPT, our findings emphasized the need for “confrontation,” “relatives’ involvement in abstinence and interventions,” “having structure and contact,” and “having a measure of control and a deterrent to drink” to reduce problematic alcohol use in older adults, according to relatives. Furthermore, perspectives of professionals and older adults should be considered alongside for a complete overview of working elements and mechanisms.

## Supplementary Information

Below is the link to the electronic supplementary material.

Supplementary file1 (DOCX 20.3 KB)

Supplementary file2 (DOCX 21.0 KB)

## Data Availability

No data are available.

## Declarations

This study received approval by the Ethics Review Board (RP508) of Tilburg University.
