# Supplementary material for: Relatives’ Perspectives on What Works to Reduce Problematic Alcohol Use in Older Adults: A Realist Evaluation
Source: Int J Ment Health Addict. 2025 Jul 14;24(3):2608–26. doi: 10.1007/s11469-025-01511-4 (PMC13246818; doi:10.1007/s11469-025-01511-4)
Supplement: Supplementary file 1 — Supplementary file1 (DOCX 20.3 KB) [file 11469_2025_1511_MOESM1_ESM.docx]

**COREQ (COnsolidated criteria for REporting Qualitative research) Checklist**

A checklist of items that should be included in reports of qualitative research

| **Topic** | **Item No.** | **Guide Questions/ Description** | **Author Responses** |
| --- | --- | --- | --- |
| **Domain 1: Research team and reflexivity** | | | |
| *Personal Characteristics* | | | |
| Interviewer/facilitator | 1 | Which author/s conducted the interview or focus group? | Fieke van den Bulck |
| Credentials | 2 | What were the researcher’s credentials? E.g. PhD, MD | MSc |
| Occupation | 3 | What was their occupation at the time of the study? | PhD |
| Gender | 4 | Was the researcher male or female? | Female |
| Experience and training | 5 | What experience or training did the researcher have? | Conducted interviews as part of her PhD |
| *Relationship with participants* | | | |
| Relationship established | 6 | Was a relationship established prior to study commencement? | Yes |
| Participant knowledge of the interviewer | 7 | What did the participants know about the researcher? e.g. personal goals, reasons for doing the research | Created relationship during preparation for the research study. Participants had knowledge of the reasons for doing the research. |
| Interviewer characteristics | 8 | What characteristics were reported about the inter-viewer/facilitator? e.g. Bias, assumptions, reasons and interests in the research topic |  |
| **Domain 2: Study design** | | | |
| *Theoretical framework* | | | |
| Methodological orientation and Theory | 9 | What methodological orientation was stated to underpin the study? e.g. grounded theory, discourse analysis, ethnography, phenomenology, content analysis | Interviews with a realist evaluation approach |
| Sampling | 10 | How were participants selected? e.g. purposive, convenience, consecutive, snowball | Purposive and convenience sample |
| Method of approach | 11 | How were participants approached? e.g. face-to-face, telephone, mail, email | Email or telephone |
| Sample size | 12 | How many participants were in the study? | 14 |
| Non-participation | 13 | How many people refused to participate or dropped out? Reasons? | Because recruitment of relatives was conducted through professionals and older adults, we cannot provide an exact number for this. |
| *Setting* | | | |
| Setting of data collection | 14 | Where was the data collected? e.g. home, clinic, workplace | The interviews with relatives were conducted face to face, online, or by telephone |
| Presence of non-participants | 15 | Was anyone else present besides the participants and researchers? | No |
| Description of sample | 16 | What are the important characteristics of the sample? e.g. demographic data, date | See Table 1 in manuscript |
| *Data collection* | | | |
| Interview guide | 17 | Were questions, prompts, guides provided by the authors? Was it pilot tested? | An interview guide was used, see Supplementary material |
| Repeat interviews | 18 | Were repeat interviews carried out? If yes, how many? | No |
| Audio/visual recording | 19 | Did the research use audio or visual recording to collect the data? | Audio recordings for Skype and telephone, visual recordings for Microsoft Teams interviews |
| Field notes | 20 | Were field notes made during and/or after the interview or focus group? | Yes |
| Duration | 21 | What was the duration of the interviews or focus group? | The interviews lasted from 32.6 to 70.1 minutes |
| Data saturation | 22 | Was data saturation discussed? | Interviews were conducted until data saturation occurred |
| Transcripts returned | 23 | Were transcripts returned to participants for comment and/or corrections? | No |
| **Domain 3: analysis and findings** | | | |
| *Data analysis* | | | |
| Number of data coders | 24 | How many data coders coded the data? | Two |
| Description of the coding tree | 25 | Did authors provide a description of the coding tree? | The code tree was based on the initial program theory, that encompassed the CEMO configurations |
| Derivation of themes | 26 | Were themes identified in advance or derived from the data? | Themes were derived from data |
| Software | 27 | What software, if applicable, was used to manage the data? | Atlas.TI |
| Participant checking | 28 | Did participants provide feedback on the findings? | No |
| *Reporting* | | | |
| Quotations presented | 29 | Were participant quotations presented to illustrate the themes/findings?  Was each quotation identified? e.g. participant number | Yes, and each quotation is identified |
| Data and findings consistent | 30 | Was there consistency between the data presented and the findings? | Yes |
| Clarity of major themes | 31 | Were major themes clearly presented in the findings? | Yes, see Table 2 in manuscript |
| Clarity of minor themes | 32 | Is there a description of diverse cases or discussion of minor themes? | Yes, see Table 2 in manuscript |

Developed from: Tong A, Sainsbury P, Craig J. Consolidated criteria for reporting qualitative research (COREQ): a 32-item checklist for interviews and focus groups. *International Journal for Quality in Health Care*. 2007. Volume 19, Number 6: pp. 349 – 357
